# Access to antibiotics: a safety and equity challenge for the next decade

**DOI:** 10.1186/2047-2994-2-1

**Published:** 2013-01-10

**Authors:** Jean Carlet, Didier Pittet

**Affiliations:** 1 World Alliance Against Antibiotic Resistance (WAAR), 9 rue de la Terrasse, 94000 Creteil, France; 2 Hôpital St Joseph, 185 rue Raymond Losserand, 75014, Paris, France; 3 Infection Control Programme and WHO Collaboration Centre on Patient Safety, University of Geneva Hospitals and Faculty of Medicine, 4 Rue Gabrielle Perret-Gentil, Geneva, Switzerland

**Keywords:** Antibiotics, Antimicrobial resistance, Antimicrobial resistance surveillance, Antibiotics – use, Multidrug-resistant organisms

## Abstract

Bacterial resistance to antibiotics is increasing worldwide in healthcare settings and in the community. Some microbial pathogens have become resistant to multiple antibiotics, if not all presently available, thus severely compromising treatment success and contributing to enhanced morbidity, mortality, and resource use. The major driver of resistance is misuse of antibiotics in both human and non-human medicine. Both enhanced access and restricted use in many parts of the world is mandatory. There is an urgent need for an international, integrated, multi-level action to preserve antibiotics in the armamentarium of the 21^st^ century and address the global issue of antimicrobial resistance.

## Background

Antibiotics are one of the most important discoveries in medicine and have saved millions of lives. The current paradoxical scandal is that although most resource-limited countries still lack easy access to antibiotics, their overuse and abuse is rife in developed nations and the cause of accelerating rates of resistance development. A global two-pronged approach is urgently needed to promote facilitated access and to alert the scientific and lay community to the dangers of misuse, potentially leading to a severe lack of therapeutic agents to treat infections in the near future.

Access to antibiotics is a major concern in many countries worldwide [1-4]. Reasons include the very low economic status of a large number of nations, inappropriate use, the high cost of the most recent and efficacious antibiotics, extensive “over the counter” usage, an increasing number of counterfeit drugs, and a dramatic increase in antimicrobial resistance (AMR). Importantly, countries with only a few antibiotics available and a high number of deaths from infection are not protected from antibiotic resistance [5,6]. Great strides have been made towards the eradication of some pandemic infections, such as malaria, tuberculosis, and human immunodeficiency virus (HIV)/acquired immunodeficiency syndrome (AIDS), in particular due to the generous support of the Global Fund, UNITAID, The Bill & Melinda Gates Foundation, and other large international funding organizations. However, infections due to common microorganisms responsible for more widely-spread diseases still represent an extremely high overall burden worldwide, such as pneumonia, gastrointestinal infections, and meningitis.

### Resistance in healthcare

Antibiotic resistance in healthcare facilities is a very serious concern in most developed and developing nations. Only a few Scandinavian countries and the Netherlands have had the strategic vision to limit the invasion of their country by resistant strains through the implementation of very active programmes. Somewhat ironically, some bacteria, particularly Gram-negatives, are now resistant to almost every available antibiotic, including the most recent generation [7]. Some are still susceptible to colistin, a 50-year-old and rather toxic compound, almost forgotten until recently or only used in resource-limited countries [8]. Late-onset ventilator-associated pneumonia is treated empirically with colistin in the intensive care units of several countries but, unfortunately, *Pseudomonas aeruginosa* and *Acinetobacter* spp. resistance has been described [9]. The worst case scenario for the future is that an unknown number of patients might die because active antibiotics are no longer available. This is unacceptable, although far less unacceptable than the lack of access to antibiotics in many countries.

### Community-acquired resistance

Resistance to antibiotics in the community is an emerging problem in developed and developing countries and possibly even more frightening. The best examples are community- acquired methicillin-resistant *Staphylococcus aureus* (MRSA), particularly in the USA [10], *Escherichia coli* or *Klebsiella pneumonia*-producing extended-spectrum beta-lactamases (ESBL) [11] and, more recently, carbapenemases [12]. The risk is that carbapenems could replace cephalosporins or broad-spectrum penicillins for the empiric treatment of community-acquired diseases, such as pyelonephritis or intra-abdominal infections, where our lifelong “partners”, enterobacteriacae, are involved. Carbapenems are the main antibiotics that remain efficient for the treatment of hospital-acquired ESBL infections and represent our last line of defence in many regards. Many carbapenemases have already been described, notably in *E. coli* and *K. pneumonia*[12], and this class of antibiotics is in real danger and must be protected. To achieve this, carbapenem use must be regulated and restricted to severe infections treated with specialist input or in consultation with a clinical infectious diseases’ specialist. Combinations of beta-lactams with beta-lactamases inhibitors are likely to be helpful in the future [13,14].

We have available a good list of new compounds highly active against Gram-positive bacteria, but only very few active against multi-resistant Gram-negatives in the pipeline. This constitutes a critical safety issue worldwide and is certainly far more important in terms of potential impact than the H1N1 influenza pandemic (25,000 versus 14,000, respectively, in Europe in 2009) [15,16]. The reality is that both short- and long-term international reactions to such a threat have been too weak and too slow. We have been unable or unwilling to modify global behaviour towards antibiotic use, thus preparing a very dark future for generations to come.

### Rethinking current clinical practice

In the critically ill, particularly for septic shock, recent international guidelines strongly encourage clinicians to start broad spectrum antibiotics immediately, if possible within the first hour of admission [17]. In many institutions, delaying the start of antibiotics to treat community-acquired pneumonia is considered as an indicator of poor quality of care. Such measures make sense and possibly save lives [18]. However, the use of such indicators and the desire to meet required targets may have unintended consequences or harm. Therefore, clinicians should ensure that the diagnosis is robust to prevent unnecessary treatment with broad spectrum antibiotics. All treatment should be subject to review after two or three days and new antibiotics should require the prior input of an infection specialist [19]. In many cases, antibiotics can be stopped at days two or three or changed to narrower spectrum compounds. If combination therapy has been used empirically, subsequent reversion to monotherapy is possible in most cases. The term “de-escalation” is sometimes used to describe such a strategy, but there are two prerequisites for success. First, clinicians and microbiologists must be encouraged to obtain appropriate microbiological samples before starting empiric therapy to streamline the antibiotic strategy, particularly in the critically ill. Second, clinicians need to feel comfortable about the decision to de-escalate, even when initial therapy was effective. The motto “don’t change a winning team” is certainly not applicable to antibiotic therapy. Microorganisms represent very special therapeutic targets ― a living and respectful target. Similar to many antibiotics, bacteria have been present in the environment long before man and due respect is essential, at least from an ecological standpoint.

In some European countries, physicians feel at ease to change initial empiric antibiotic therapy even when it works, thus following recommendations issued in recently published guidelines [20]. Prescribers need help, not blame. National guidelines have been elaborated over the past few years [21,22], including for veterinary medicine [23], as well as many reports on the prevention of AMR spread [21,24,25]. However, since the report of an international task force meeting in 1995 [26], no truly coordinated, international efforts have been made to tackle the global issue of AMR and access to antibiotics, apart from specific diseases such as malaria, tuberculosis, and HIV/AIDS.

### Developing a collective conscience

Limiting antibiotic consumption comprises a certain degree of risk. Some diseases, such as post-streptococcal infections could reappear. Some infections, like meningitis, might be more frequent if patients with high fever are no longer treated with antibiotics. Risks must be evaluated, carefully followed, and balanced against the risk of resistance. National public health agencies and international organizations, such as the World Health Organization (WHO), must assume their responsibility and give clear recommendations, while taking such risks into account. There are additional philosophical and ecological reasons to focus on equity and access to antibiotics. Most antibiotics, like animals and plants, are natural products belonging to “mother nature” and humanity. Part of our collective ecological and moral duty is not only to protect them, but also to make them available to every human being. Equity is an integral part of quality and safety [27].

AMR is considered an extremely serious issue by the WHO Patient Safety programme. Its recent publication entitled "The evolving threat of antimicrobial resistance - options for action" [28] examines the experiences of governments and health facilities/providers with implementing some of the recommendations of the 2001 WHO Global Strategy for Containment of Antimicrobial Resistance. It reflects upon lessons learned during the past decade and remaining gaps, while drawing attention to areas where knowledge is still lacking and urgent action needed. One of the programme strands, African Partnerships for Patient Safety, includes the prevention of healthcare-associated infections and access to antimicrobials as it is clearly established that developing countries have very high rates of nosocomial infections and AMR [5]. The precise burden of non-access to antibiotics is unknown and these data are urgently needed. If we do not undertake this global, international, integrated action now, future generations will certainly never forgive us for having been so weak and passive to meet this challenge.

## Competing interests

The authors declare that they have no competing interests.

## Authors’ contributions

JC and DP contributed equally to the concept, drafting and critical revision of the manuscript. Both authors approved the final version submitted for publication.
